# Immunohistochemistry on a panel of Emery–Dreifuss muscular dystrophy samples reveals nuclear envelope proteins as inconsistent markers for pathology

**DOI:** 10.1016/j.nmd.2016.12.003

**Published:** 2017-04

**Authors:** Phu Le Thanh, Peter Meinke, Nadia Korfali, Vlastimil Srsen, Michael I. Robson, Manfred Wehnert, Benedikt Schoser, Caroline A. Sewry, Eric C. Schirmer

**Affiliations:** a Wellcome Trust Centre for Cell Biology, University of Edinburgh, Edinburgh, UK; b Friedrich-Baur-Institute, Ludwig Maximilian University, Munich, Germany; c Institute of Human Genetics, University of Greifswald, Greifswald, Germany; d Dubowitz Neuromuscular Centre, Great Ormond Street Hospital for Children NHS Trust, London, UK

**Keywords:** Emery–Dreifuss muscular dystrophy, Nuclear envelope, Muscle biopsy, Nuclear envelope transmembrane protein

## Abstract

Reports of aberrant distribution for some nuclear envelope proteins in cells expressing a few Emery–Dreifuss muscular dystrophy mutations raised the possibility that such protein redistribution could underlie pathology and/or be diagnostic. However, this disorder is linked to 8 different genes encoding nuclear envelope proteins, raising the question of whether a particular protein is most relevant. Therefore, myoblast/fibroblast cultures from biopsy and tissue sections from a panel of nine Emery–Dreifuss muscular dystrophy patients (4 male, 5 female) including those carrying emerin and FHL1 (X-linked) and several lamin A (autosomal dominant) mutations were stained for the proteins linked to the disorder. As tissue-specific nuclear envelope proteins have been postulated to mediate the tissue-specific pathologies of different nuclear envelopathies, patient samples were also stained for several muscle-specific nuclear membrane proteins. Although linked proteins nesprin 1 and SUN2 and muscle-specific proteins NET5/Samp1 and Tmem214 yielded aberrant distributions in individual patient cells, none exhibited defects through the larger patient panel. Muscle-specific Tmem38A normally appeared in both the nuclear envelope and sarcoplasmic reticulum, but most patient samples exhibited a moderate redistribution favouring the sarcoplasmic reticulum. The absence of striking uniform defects in nuclear envelope protein distribution indicates that such staining will be unavailing for general diagnostics, though it remains possible that specific mutations exhibiting protein distribution defects might reflect a particular clinical variant. These findings further argue that multiple pathways can lead to the generally similar pathologies of this disorder while at the same time the different cellular phenotypes observed possibly may help explain the considerable clinical variation of EDMD.

## Highlights

• Altered distribution of EDMD-linked proteins is not a general characteristic of EDMD.
• Tissue-specific proteins exhibit altered distributions in some EDMD patients.
• Variation in redistributed proteins in EDMD may underlie its clinical variability.

## 1 Introduction

Emery–Dreifuss muscular dystrophy (EDMD) typically presents in early childhood with slow progression, though adult onset also occurs [1], [2]. Three defining features of this disorder include early contractures of the elbows and Achilles' tendons in the absence of major muscular defects, progressive wasting of the lower leg and upper arm muscles and cardiac conduction defect [3]. All these features are variable in clinical presentation: while typical patients remain ambulatory, severe cases require wheelchairs. Likewise, cardiac defects do not always present, but complete heart block can occur in the most severe cases. Conduction defects can also present in the absence of prior muscular involvement [2] and female carriers of the X-linked form can develop cardiac problems [4]. Even within the same family, the same mutation can yield highly variable clinical presentation amongst family members [5], [6], [7].

With this clinical variability it was not surprising to find that EDMD is also genetically variable. Mutations in 8 nuclear envelope proteins account for ~47% of patients. The vast majority of mutations are X-linked in *EMD* (encoding emerin) [8] and autosomal dominant in *LMNA* (encoding lamins A and C) [9] though more rare autosomal recessive *LMNA* mutations also occur [10]. Lamin A is a nuclear intermediate filament protein that lines the inner surface of the nuclear envelope while emerin is a nuclear envelope transmembrane protein (NET). Roughly 3% of patients are linked to mutations in 5 other NETs: *TMEM43*, *SYNE1*, *SYNE2*, *SUN1* and *SUN2* [11], [12], [13]. The remaining 3% of known mutations are linked to *FHL1* (encoding Four and a half LIM domain 1) [14]. *FHL1* has many splice variants that have multiple cellular localisations including muscle z-bands and the nucleus, but FHL1B targets also to the nuclear envelope [15]. *FHL1* is also linked to other myopathies such as X-linked myopathy with postural muscle atrophy (XMPMA) [16] and deletion in mice leads to muscle hypertrophy [17]. The strong nuclear envelope links for nearly half of all cases raises the possibility of a common pathway at the nuclear envelope affected in EDMD.

The principal mechanisms proposed to explain how nuclear envelope disruption can yield pathology are genome misregulation, mechanical instability and failure of stem cell maintenance – all potentially leading to impaired differentiation [18], [19], [20], [21], [22]. However, it is unclear how mutations in these widely expressed proteins can cause this muscle-specific disorder. One proposed model is that muscle-specific partners that function in complexes with these widely expressed nuclear envelope proteins might mediate the muscle-specific pathologies. Several candidates were identified by proteomics of muscle nuclear envelopes [23]. WFS1, Tmem214 and Tmem38A/TRIC-A were identified only in muscle out of several tissues separately analysed by proteomics for nuclear envelopes [24]. NET5/Samp1 was found in nuclear envelopes from other tissues, but has a muscle-specific splice variant [25]. Several of these are candidates for mechanical functions due to implied connections to the cytoskeleton: NET5/Samp1, WFS1 and Tmem214 localise to the mitotic spindle [23], [26] and NET5/Samp1 knockdown dissociates centrosomes from the NE [26]. As the centrosome organises microtubule networks and cell polarity, disrupting its association with the nuclear envelope could result in contractile defects in myofibres. Tmem214 additionally tracked with microtubules on the nuclear surface [23] and thus could influence nuclear rotation and migration to the edges of the myofibres. WFS1 also has a separate function shared by Tmem38A/TRIC-A in genome organisation and regulation of gene expression during myogenesis and knockout of these two muscle NETs together with a third with the same function completely blocked myotube differentiation [27]. Tmem38A/TRIC-A separately contributes to the regulation of calcium ion transport [28], [29], [30], [31] and thus could affect either muscle contraction or signalling at the nuclear envelope. That some of these muscle-specific NETs had overlap in their functions further supports the possibility of their working in a common pathway towards EDMD pathophysiology.

We postulated that if a central mechanism at the NE underlies EDMD pathology through disruption of a functional complex then components of that complex might redistribute away from the NE. Early studies reported that emerin depends on lamin A for its localisation to the nuclear envelope [22], [32] and that lamin EDMD mutation L530P and mutation R377H from a family with dilated cardiomyopathy combined with specific quadricep muscle myopathy similarly yield a notable loss of emerin at the nuclear envelope in tissue culture cells [33], [34]. Emerin also redistributed away from the NE in fibroblasts from a patient with an EDMD mutation in nesprin, another NET. The single nesprin 2β (*SYNE2*) T89M mutation resulted in redistribution of emerin to the cytoplasm while this same mutation combined with a nesprin 1α (*SYNE1*) V572L mutation resulted in redistribution of emerin to the polar cap [13]. The nesprin double mutation yielded a slightly different redistribution of emerin to the plasma membrane in actual muscle sections [13]. Correspondingly, nesprin redistributed away from the NE in fibroblasts from a patient with an emerin EDMD g.631delTCTAC mutation that results in loss of exon 6 [13]. Interestingly, in one of these studies two cardiomyopathy lamin A mutants studied (L85R and N195K) had variable emerin mislocalisation phenotypes while a lipodystrophy mutation (R482W) exhibited no altered localisation [34], suggesting that NET mislocalisation might be a specific feature of nuclear envelope linked muscle disorders.

No study has systematically tested for the mislocalisation of the wider range of EDMD-linked proteins in a panel of patients covering the genetic spectrum of EDMD. Here we stained a wide panel of EDMD muscle biopsy sections and cultured myoblast/fibroblast cultures from biopsies with a panel of antibodies to the EDMD-linked proteins. To investigate potential muscle-specific NET involvement in mechanisms to generate the pathology of the disorder, we also stained these samples with antibodies against the muscle-specific NETs NET5/Samp1, WFS1, Tmem214 and Tmem38A. We find that neither emerin nor lamin A nor any of the other NETs are uniformly altered in all patient samples. However, nesprin 1, SUN2, and several muscle-specific NETs exhibited unusual distribution patterns in a subset of samples. These findings indicate that there are likely to be multiple pathways leading to EDMD pathology and suggest the possible involvement also of these muscle-specific NETs in the disorder.

## 2 Materials and methods

### 2.1 Patient materials and ethics

Primary human myoblast/fibroblast cultures (Table 1) and muscle biopsies for sectioning (Table 2) were obtained from either the Centre for Inherited Neuromuscular Disease (CIND) in Oswestry through C.S., the MRC Centre for Neuromuscular Disorders Biobank (CNDB) in London, or the Muscle Tissue Culture Collection (MTCC) at the Friedrich-Baur-Institute (Department of Neurology, Ludwig-Maximilians-University, Munich, Germany). All control and patient materials were obtained with informed consent of the donor at the CIND, the CNDB or the MTCC. Ethical approval for this particular study was obtained from the West of Scotland Research Ethics Service (WoSRES) with REC reference 15/WS/0069 and IRAS project ID 177946.

### 2.2 Cell maintenance

Primary human myoblast/fibroblast cultures obtained from patient biopsy were maintained in skeletal muscle cell growth medium (PromoCell C-23060). Cells were kept from reaching confluency to avoid differentiation. For myoblast differentiation into myotubes, the primary human myoblasts in the cultures were differentiated using a matched differentiation medium (PromoCell C-23061). C2C12 cells were maintained in DMEM with 20% Foetal calf serum and antibiotics. All cells were maintained at 37 °C in a 5% CO_2_ incubator.

### 2.3 Antibodies for immunostaining

Antibodies were obtained from multiple sources and used at several different dilutions (Table 3). Tmem38A and NET5/Samp1 were affinity purified against the protein fragment/peptide used in their generation. The antibody baits were dialysed out of their storage buffer into PBS and coupled to Affi-Gel matrix. Antibodies were bound to the column from serum, eluted with 200 mM Glycine pH 2.3 and the buffer was immediately exchanged using spin concentrators to PBS containing 25% glycerol. All secondary antibodies were donkey minimal cross-reactivity Alexafluor-conjugated from Invitrogen except for those used for Western blot, which were also donkey minimal cross-reactivity IRDye^®^-conjugated from LI-COR.

### 2.4 Western blotting

Protein samples were separated by SDS–PAGE, transferred onto nitrocellulose membranes (Odyssey 926-31092) and blocked 30 min in Western blot blocking buffer: 5% milk powder and 0.05% Tween-20 in TBS (25 mM Tris-Base, 137 mM NaCl, 2.7 mM KCl). Membranes were incubated with primary antibodies in Western blot blocking buffer overnight at 4 °C. Six washes in TBS-0.05% Tween-20 were then followed by incubation with the secondary antibodies for 60 min at room temperature. After another 6 washes in TBS-0.05% Tween-20 antibody signals were detected on a LI-COR Odyssey Quantitative Fluorescence Imager.

### 2.5 Immunofluorescence

Adherent cells grown on uncoated coverslips were washed in PBS prior to fixation with −20 °C 100% methanol and immediately stored at −20 °C. Methanol fixation was used because it improved epitope accessibility for the antibodies and should precipitate membrane proteins at location rather than wash them away. Prior to staining cells were incubated 10 min in TBS-0.1% Tween-20. Coverslips were blocked in 1X immunofluorescence blocking buffer (1% Horse Serum, 1% Foetal Calf Serum, 0.1% Bovine Serum Albumin in PBS pH 7.2) for 20 min at RT and incubated with primary antibodies. Following 3 washes in TBS-0.1% Tween-20, coverslips were incubated with secondary antibodies and 4 µg/ml 4,6-diamidino-2 phenylindole, dihydrochloride (DAPI). Coverslips were extensively washed in PBS or TBS-0.1% Tween-20 over 30 min and mounted with VectaShield (Vector Labs).

Muscle biopsies were mounted on cork in OCT mounting medium (ThermoFisher Scientific LAMB/OCT) and frozen in isopentane cooled in liquid nitrogen. 10 µm sections were cut using a Leica CM1900 cryostat and collected on SuperFrost Plus (VWR) slides, placed immediately on dry ice and stored at −80 °C. Sections were brought to RT before staining. Using a PAP hydrophobic marker pen (Daido Sangyo), a working area was drawn around each section and the sections were washed in immunofluorescence blocking buffer for 30 min. Sections were incubated in primary antibodies overnight at 4 °C in a humidified chamber and then washed 3 × 5 min using TBS-0.1% Tween-20. Secondary antibodies were applied for 1 h, then removed gently by blotting with tissue paper and DAPI applied for 10–15 min. Sections were then washed 3 × 10 min in TBS-0.1% Tween-20 and 1 × 10 min in TBS. Excess liquid was blotted off carefully using tissue paper, a drop of Vectashield added and a coverslip applied.

### 2.6 Microscopy and analysis

Images were acquired on a Nikon TE-2000 widefield microscope using a 1.45 NA 100× objective, Sedat quad filter set, PIFOC z-axis focus drive (Physik Instruments) and a CoolSnapHQ High Speed Monochrome CCD camera (Photometrics) run by Metamorph image acquisition software. Widefield images are mostly shown, but for Fig. 5 deconvolved images are shown. For these, z-stacks acquired at intervals of 0.2 µm from the 1 µm above to 1 µm below the imaged nucleus were deconvolved using AutoQuant X3.

## 3 Results

### 3.1 Distribution of EDMD-linked proteins in cultured patient muscle cells

Several earlier reports presented data showing that emerin, nesprins and lamin A/C staining, normally concentrated at the nuclear envelope, was aberrant variously in lamin A knockout cells and cells expressing certain EDMD lamin A, emerin and nesprin mutations [13], [22], [32], [34]. However, typically only a single patient mutation was tested and only lamin A/C and a few NETs were tested for any given sample, though EDMD has now been linked to 8 different nuclear envelope proteins. To determine if any particular one of these proteins is recurrently defective in its intracellular distribution we stained a panel of 3 control and 8 EDMD patient myoblast/fibroblast cultures (Table 1) for emerin, lamin A/C, nesprin 1, nesprin 2, SUN1, SUN2, and FHL1. Although images are likely to contain a mixture of myoblasts and fibroblasts, we expect that the majority of cells are likely to be myoblasts as staining cultures for 4 of the patients with desmin antibodies revealed 78%, 56%, 100% and 78% of DAPI-stained nuclei in desmin positive cells respectively for patients P1, P5, P6 and P7. All stainings were done in parallel and all images were taken with the same exposure times and microscope software settings.

**Fig. 1:**
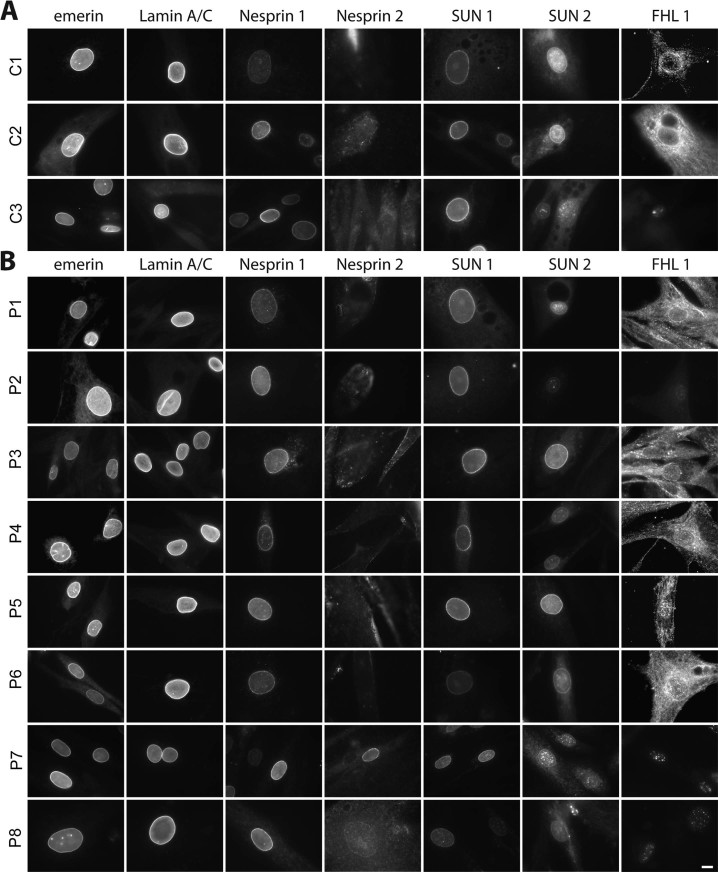
Staining of control (A) and patient (B) myoblast/fibroblast cultures with antibodies to EDMD-linked proteins. The patient and control descriptions are given in Table 1 and the antibodies used are described in Table 3. Most nuclear envelope proteins gave strong crisp nuclear rim staining in both control and patient groups. Though some EDMD-linked patient cells exhibited partial mislocalisation, no linked proteins exhibited uniform mislocalisations in the wide range of patient mutations investigated. The nesprin 2 antibodies do not stain well in the myoblast/fibroblast cultures; so these were retested on differentiated cells in Fig. 2. Widefield images are shown. Scale bar, 10 µm.

This panel included patients with lamin A/C-, emerin-, and FHL1-linked disorder. Surprisingly, emerin, despite previous reports of its aberrant distribution, exhibited strong nuclear envelope staining with a crisp rim of fluorescence at the nuclear perimeter (nuclear rim) in all patient cells indistinguishable from the control cells (Fig. 1). Patient P3 was a female with a heterozygous truncation mutation in the X chromosomal gene encoding emerin. Though unusual for a female carrying an emerin mutation to have a muscle phenotype, the affected father also carried the emerin mutation. Patient P3 expressed full-length emerin in a subset of cells, excluding uneven X-inactivation, and, together with the father's earlier presentation than his affected uncles, this possibly indicates an additional unknown mutation [35]. Here this subset of emerin-positive cells exhibited a moderately weaker staining compared to that in other patients. While some emerin accumulation in the ER appeared in patients P2 and P5, it was not more than for control C2, and this control had more ER accumulation than other EDMD patient cells. Thus, any minor differences in emerin distribution were within the same breadth of such differences exhibited by the control group.

No visible differences were observed for lamin A/C staining between the patient and control cells and even within each set, unlike emerin where both some control and some patient cells exhibited minimal ER accumulation (Fig. 1). The image selected for control C3 was chosen because the cell was smaller and had more nucleoplasmic lamin staining, likely due to being at an earlier cell cycle stage. Cells shortly after mitosis characteristically have larger nucleoplasmic lamin pools because the lamins remaining from the previous cell cycle have not fully reassembled and this pool disappears as nuclear volume increases. None of the larger or smaller cells from the patients had more nucleoplasmic lamin accumulation than this control, further underscoring the fact that any minor visible differences can be discounted.

**Fig. 2:**
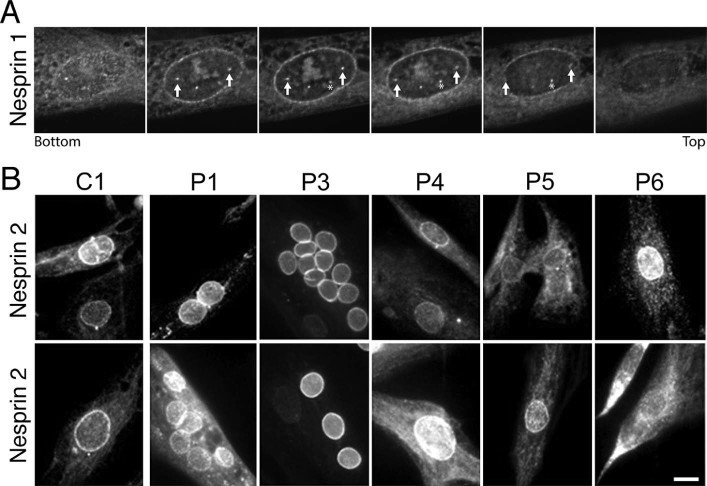
Staining of myoblast/fibroblast cultures and differentiated myotubes with nesprin antibodies. (A) To determine whether intranuclear spots staining with nesprin antibodies reflected invaginations of the nuclear envelope as opposed to possible degradation/cleavage products, z-series images of cells stained with nesprin 1 antibodies were taken every 0.2 µm. In the images shown the arrows and asterisk point to different intranuclear spots that can be traced through the different focal planes to invaginations from the nuclear membrane. (B) Staining after induction of differentiation into myotubes for nesprin 2. Large multinucleated myotubes were not obtained from all patients; however, changes in morphology such as elongating of the cell body or much larger cells indicative of cell cycle withdrawal were generally evident and rim staining could be readily observed compared to the very poor rim staining in the myoblast/fibroblast cultures stained in Fig. 1. Multiple images for each patient are shown to show the variability between cells. Widefield images are shown. Scale bar, 10 µm.

For the other NETs and FHL1 there was greater variation amongst samples, but in nearly all cases a similar range of variation was observed for the controls (Fig. 1). For example, in multiple controls nesprin 1 staining was variable in intensity at the nuclear membrane compared from cell to cell in the same field (Fig. 1A). Also some control cells exhibited spotty intranuclear staining while others did not, with similar variation observed also in the patient cells (Fig. 1B). Analysis of this intranuclear staining in z-series indicated that it reflects invaginations of the nuclear membrane (Fig. 2A). Roughly half of the control cells also exhibited some punctate staining in the nucleoplasm (Fig. 1A), most likely due to invaginations, but possibly also soluble splice variants (the antibody used, MANES1E(8C3), was generated to full-length nesprin1-α). Within the patient population similar variation was observed in overall intensity, relative rim intensity and punctate areas. However, patients P3 and P4 exhibited minor staining in the ER that was not observed for either the controls or the other patients. Although this is a different specific mutation, the P3 staining is consistent with the previous report of nesprin mislocalisation with emerin EDMD mutation g.631delTCTAC resulting in loss of exon 6 [13]. This is a new observation for the P4 LMNA p.R545C mutation, but notably other lamin and the FHL1 mutant myoblast/fibroblast cultures did not exhibit similar ER accumulations; thus, this difference is not a general characteristic of EDMD. SUN2 also exhibited some ER accumulation in myoblast/fibroblast cultures from two patients, but these were different patients with lamin mutations (LMNA p.T528K and LMNA p.E358K) and some ER accumulation was also observed in the control myoblast/fibroblast cultures. In general SUN2 and FHL1 exhibited the most variable staining patterns, but as variability was also observed in the controls this may reflect effects of the cell cycle or differentiation state.

This latter issue of differentiation state is likely the reason for the poor staining of nesprin 2, which is stained well by this antibody in differentiated myofibres [36]. Notably, the one patient with clear rim staining, P6 (LMNA p.T528K), had the appearance of multiple nuclei lined up in a myotube while the weak rim staining for P7 (LMNA p.E358K) appears to reflect a senescent cell by its extremely large nucleus and spread cytoplasm. Therefore we also stained for nesprin 2 after induction of differentiation in reduced serum differentiation medium (Fig. 2). Not all patient cells differentiated efficiently into fused myotubes, perhaps due to myoblast passage number in culture or different amounts of contaminating fibroblasts. Nonetheless, a distinct rim-staining pattern could be observed in both the C1 control and all EDMD patient cells tested.

### 3.2 Distribution of muscle-specific NETs in cultured EDMD patient myotubes

As the EDMD-linked NETs are all widely expressed and known to have many binding partners, we considered that their failure to exhibit aberrant distribution patterns uniformly through the set of patient samples might reflect redundancy in the partners to retain them at the nuclear membrane. As mutations in widely expressed nuclear envelope proteins cause a much wider range of tissue-specific disorders including also lipodystrophy, dermopathy, neuropathies and bone disorders, it has been proposed that tissue-specific binding partners might mediate the tissue-specific pathologies [37]. Therefore, we postulated that muscle-specific partners might contribute to the pathology of the disorder, have fewer binding sites and be more likely to be disrupted in their distribution in patients.

**Fig. 3:**
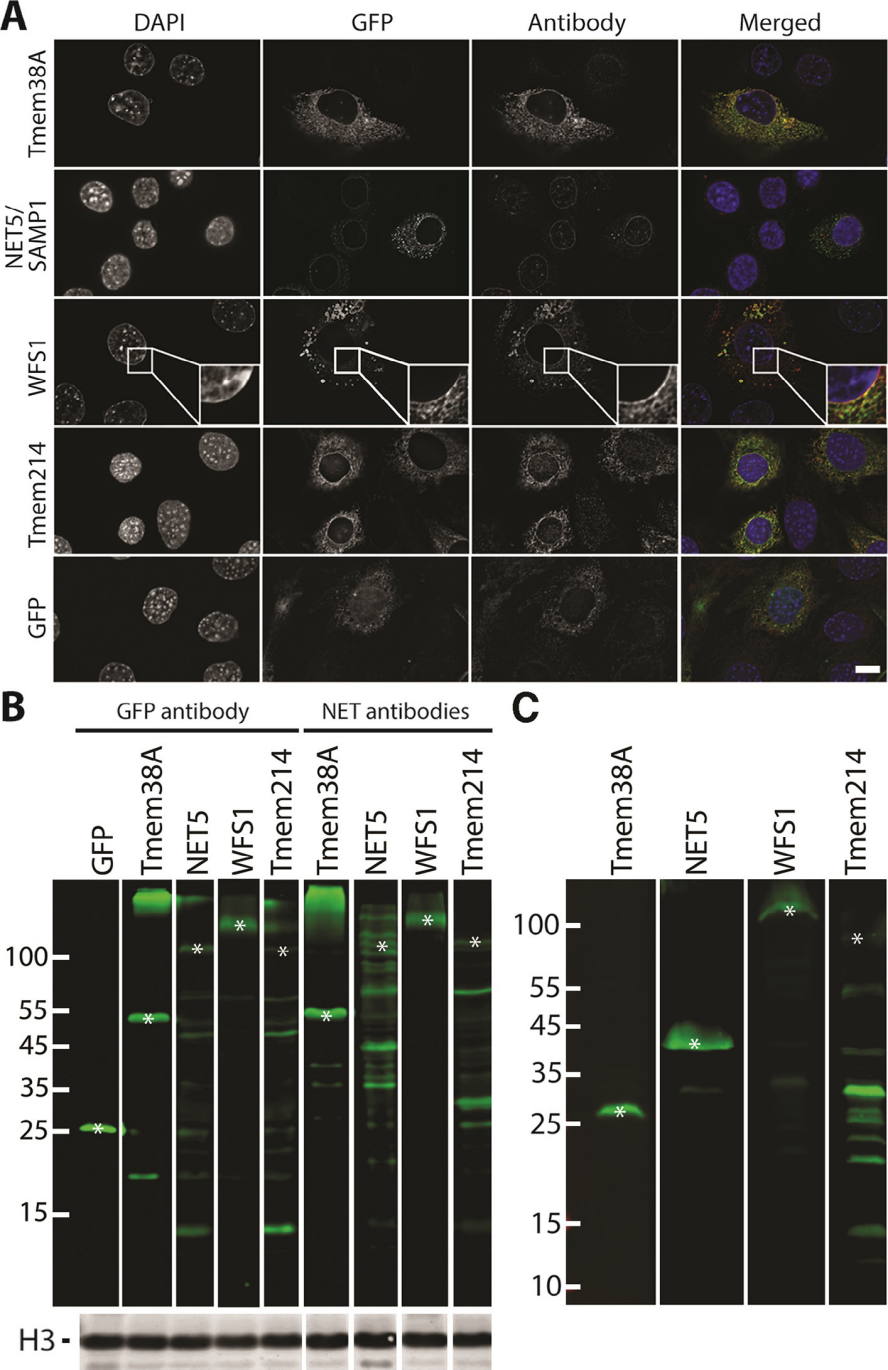
Testing of antibodies for muscle NETs. C2C12 cells were transduced with GFP fusions to the NETs or GFP alone and these cells were divided into two populations. (A) The first was used to check for co-localisation between the antibody and GFP signal for the expressed protein. The GFP signal and NET and GFP antibody signals overlapped in all cases. The antibodies used in the antibody column match the GFP fusion proteins being expressed that are labelled on the left hand side. In the case of WFS1 as the GFP signal around the nuclear rim was weaker evidently than the endogenous protein staining with the antibody a box at the rim is enlarged so that the clear co-localisation can be seen. Widefield images are shown. Scale bar, 10 µm. (B) The second population was used to generate lysates to test by Western blot. The expected size for GFP–NET fusion bands is marked by asterisks and it can be observed that the same expressed protein fused to GFP is recognised by both the GFP and muscle NET antibodies. Histone H3 (lower panel) was used as a loading control. (C) Because there is often species cross-reactivity and extra bands from degradation products of overexpressed proteins, the antibodies were also used to stain a lysate from a control human muscle biopsy. This indicated that the Tmem38A, NET5 and WFS1 antibodies are quite specific so that any protein redistribution observed in immunofluorescence experiments should be specific to the NET. Tmem214 stained additional lower molecular weight bands that might be degradation products, but could also indicate cross-reactivity with other proteins. Therefore, immunofluorescence experiments with this last antibody should be interpreted with caution.

Antibodies were obtained for Tmem38A, NET5/Samp1, WFS1 and Tmem214 and tested for their specificity. C2C12 cells were transduced with lentiviruses encoding GFP fusions to these NETs, fixed, and stained with the NET antibodies. In all cases the GFP-signal co-localised with the NET antibody signal (Fig. 3A). Notably, for NET5/Samp1 the endogenous rim staining was sufficiently stronger than the GFP-fusion that an even more pronounced rim was observed in the antibody stained sample than for the GFP signal. This is particularly apparent because some of the overexpressed exogenous GFP-fusion protein accumulated in the ER, most likely due to saturation of binding sites at the nuclear envelope. The antibodies were also tested by Western blot from lysates generated from additional cells from the same transfections (Fig. 3B). In all cases the band recognised by GFP antibodies for the muscle NET–GFP fusion was also recognised by the muscle NET antibody. Hence, all antibodies recognise the target NET. Because the antibodies were to be used to stain human cells, they were also tested on a lysate from a human control muscle biopsy (Fig. 3C). This yielded strong staining principally for just one band for the Tmem38A, NET5/Samp1 and WFS1 antibodies, indicating that they should each specifically recognise their target protein for the immunofluorescence images in subsequent figures. The Tmem214 antibody was much less clean than the other NET antibodies and so it should be understood that nuclear envelope redistributions could reflect additional proteins that it recognises as well as the Tmem214 protein.

**Fig. 4:**
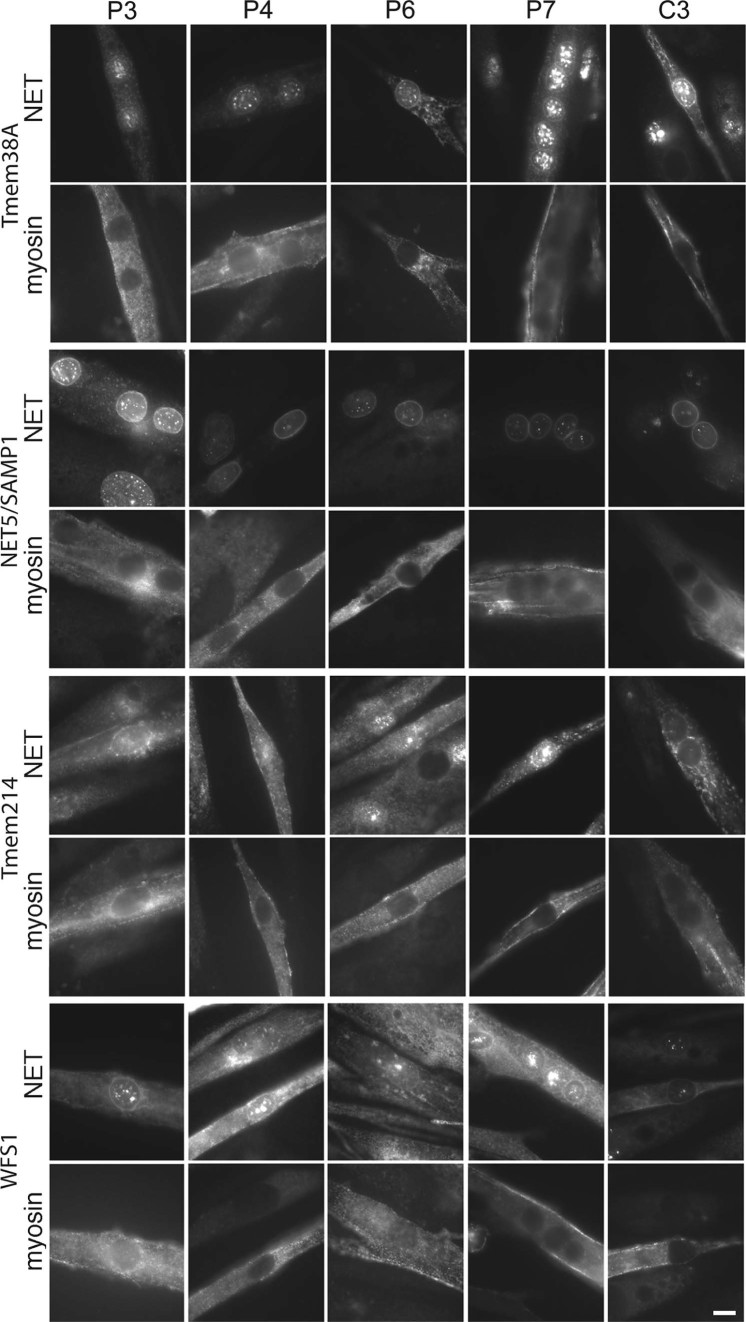
Staining of muscle NETs in patient myoblast/fibroblast cultures where myoblasts were induced to differentiate into myotubes. Antibodies to the muscle NETs listed were used to stain the control and patient cells listed. The cells were co-stained with myosin (FAST), a later differentiation marker, to identify cells that had differentiated within the population. Several EDMD patient cells exhibited more ER signal for the NETs compared with the control cells. Widefield images are shown. Scale bar, 10 µm.

Tmem38A and WFS1 are induced during muscle differentiation [27] and a muscle-specific isoform of NET5/Samp1 has been reported [25]. Therefore patient myoblast/fibroblast cultures were induced to differentiate the myoblasts into myotubes for staining. These cells were co-stained with myosin (FAST) (Fig. 4) as a marker for differentiation to distinguish cells that may have poorly differentiated due to the EDMD mutation and contaminating fibroblasts (Fig. 4). The necessity of performing this analysis in differentiated cells was highlighted in all cases by the lack of rim staining in cells lacking the red myosin (FAST) signal. A clear rim with some punctate areas inside the nucleus was observed in the C3 control for the Tmem38A antibody. Similar staining was observed for P5 but a significant loss of rim staining and strong increase in the punctate areas was visually clear for the other lamin and emerin mutations (Fig. 4, upper left panels).

NET5/Samp1 exhibited clear nuclear rim staining in all differentiated cells for both the control and EDMD patient myotubes; however, a visible relative increase in ER staining was observed for the P5 and P3 patient samples (Fig. 4, upper right panels). For Tmem214 a weak rim could be discerned in all samples except for EDMD patient sample P4 while no WFS1 rim could be discerned in EDMD patient sample P5 though much stronger ER staining was observed for patients P6 and P4. Thus, none of the muscle-specific NETs yielded a uniform redistribution phenotype in all patient samples; however, each yielded different aberrant distribution patterns in cells from distinct subsets of patients.

### 3.3 Distribution of muscle-specific NETs in EDMD patient skeletal muscle sections

**Fig. 5:**
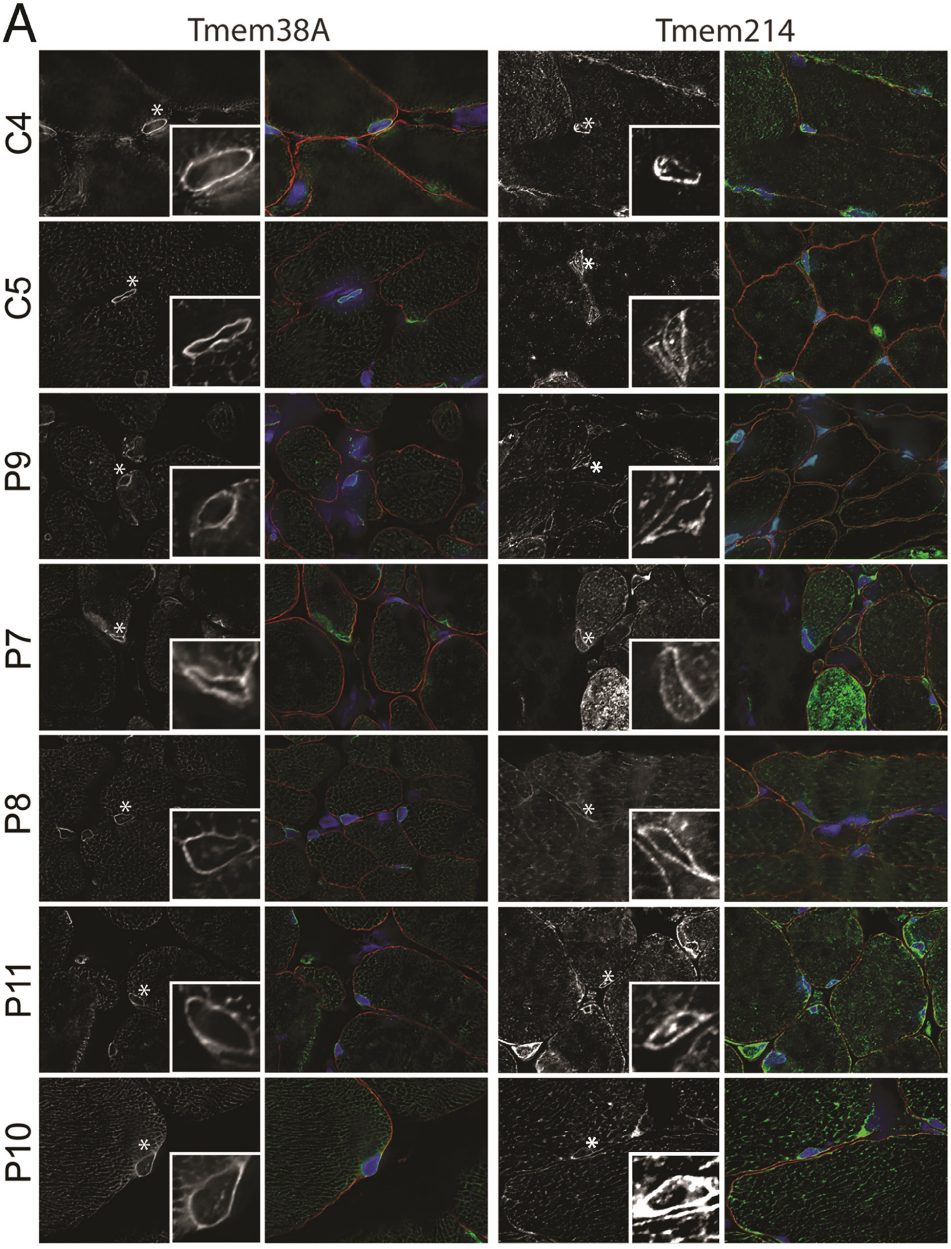

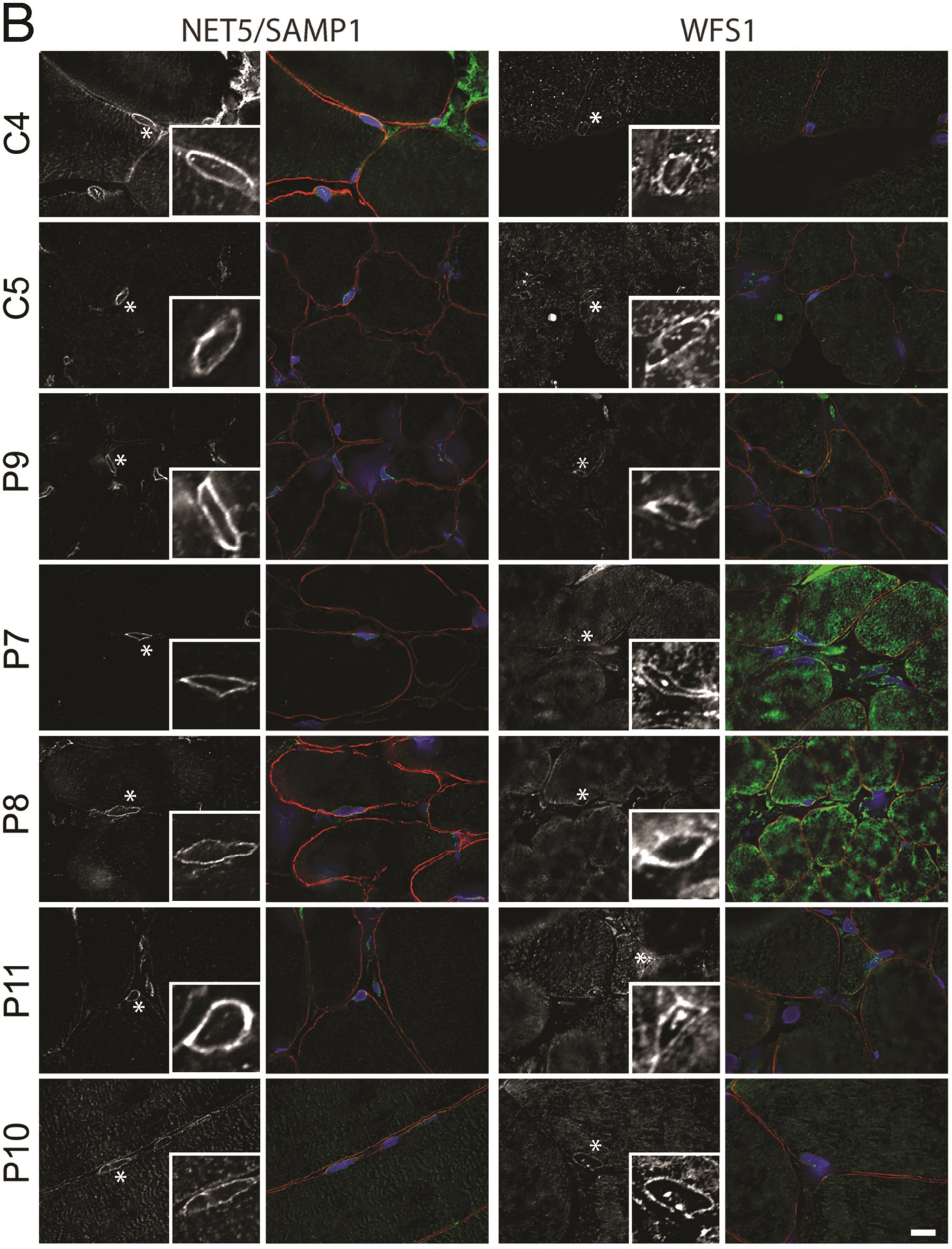
Muscle NET antibody staining in patient skeletal muscle sections. C4 and C5 are controls. The patient mutations are listed in Table 2. All sections were stained with muscle NET antibodies in parallel, images were taken using identical settings and later levels were adjusted. DNA staining for nuclei is shown in blue, the muscle NET in green, and Dystrophin staining is shown in red to delineate the sarcolemmal nuclei of myofibres from those of nerves and the vasculature. Asterisks mark the nuclei that are enlarged in the lower right boxes. Note that the levels are adjusted so the intensities are similar and they can be compared for general characteristics. (A) Tmem38A and Tmem214 antibodies. (B) NET5/Samp1 and WFS1 antibodies. Tmem38A and NET5/Samp1 distribution and intensity were much more uniform across the samples than were Tmem214 and WFS1. Deconvolved images are shown. Scale bar, 10 µm.

There are many different aspects of cultured cell growth that could potentially contribute to protein redistribution through stress effects. Some of these are difficult to control for such as pH changes and nutrient availability due to differences in growth rates between different patient cultures. Others, such as differing passage numbers from patient myoblasts/fibroblasts and thus progress towards senescence, are often unknown. Therefore, we sought to confirm these results in skeletal muscle biopsies from EDMD patients. As muscle sections contain other cell types, these were co-stained for dystrophin to delineate the plasma membrane of muscle cells and some images were chosen specifically to show that some NETs clearly only stain in the muscle nuclei and not the nuclei of these other cell types in muscle sections (Fig. 5). For example, in Fig. 5A both controls and all but patient P10 have nuclei outside fibres that are negative for Tmem38A. In Fig. 5B it is interesting that patient P8 has a nucleus outside the fibre that is negative for WFS1 while patient P11 has one outside that is positive. All images were taken at the same microscope settings and later levels were adjusted. For Tmem38A the controls C4 and C5 exhibited crisp nuclear rim staining with weaker distribution through the sarcoplasmic reticulum (Fig. 5, left top two panels). Crisp nuclear rim staining could be observed in all patient sections (Fig. 5, left lower panels); however, the relative intensity of nuclear rim to sarcoplasmic reticulum staining was notably diminished compared to the controls. Unlike differences in the cultured cells that were patient-mutation specific, this difference was observed generally.

For Tmem214 a nuclear rim stain could be observed in all samples, both control and patient; however, this time differences in the relative and absolute intensities varied between patient samples so that no generalised difference could be observed. Notably, the nuclear rim staining for this NET was much more crisp and clear than in the cultured myotubes. In patients P6 and P9 a nucleus for a cell in the space between the myofibres as delineated by dystrophin staining, possibly a capillary nucleus (Fig. 5, red), had a much stronger nuclear rim staining than the nuclei in the muscle fibres in contrast with Tmem38A staining where nuclei outside the muscle fibres were completely negative.

NET5/Samp1 stained the control nuclei very strongly against a weak background in the sarcoplasmic reticulum and this was the same for most patients. Moreover, some staining could be observed at the plasma membrane co-localised with the dystrophin membrane marker in the controls and most patients, but this was not present in patients P6 and P7. Finally, WFS1 exhibited weak staining at both the nuclear rim and sarcoplasmic reticulum in all fibres. Taking all images using the same settings the intensity of staining varied much more than for other muscle NETs, but this could reflect accessibility in the different sections as when the intensity of staining was equalised in the enlarged region boxes the character of staining was quite similar between patients. Thus in summary, Tmem38A generally appeared to have more accumulation in the sarcoplasmic reticulum in all the patients and both Tmem214 and NET5/Samp1 appeared to exhibit some differences from the controls in different subsets of patients.

## 4 Discussion

These results indicate that the previous finding of emerin redistributing away from the nuclear envelope with loss of lamin A or lamin A EDMD mutation L530P and mutation R377H from a family with dilated cardiomyopathy combined with specific quadricep muscle myopathy [22], [32], [33], [34] is not a general characteristic of AD-EDMD. Only a few patients had been tested for this before, but by comparing a wider panel of EDMD mutations it is now clear that the emerin redistribution effects are only characteristic of a subset of mutations. Notably, the use of 3 separate controls revealed that to some extent emerin redistribution can occur in cells even in the absence of EDMD mutations. Thus, the relevance of this redistribution to EDMD pathology is unclear even in the patients where it was observed. One recent study suggested a link between emerin cytoplasmic accumulation and pathology in that emerin-p.P183T assembles into oligomers that perhaps cannot pass through the peripheral channels of the nuclear pore complexes [38]. Nonetheless, unlike this particular case, most reported emerin mutations result in a loss of protein.

While the specific mutations analysed in this study and earlier studies differed, another aspect that may have contributed to redistribution phenotypes previously reported is the use of complete knockout or mutant over-expression and the use of rapidly dividing cancer cell lines. Two of the earlier studies focused on lamin knockout or loss [22], [32], but most lamin EDMD mutations are dominant, total lamin levels generally appear normal where tested, and the point mutations by prediction should not block targeting and integration into the lamin polymer. The lamin mutations analysed here included mutations in the N-terminus (p.N31del), the rod (exon 3, p.E358K), the Ig fold (p.R453W), the edge of the Ig fold (p.R545C) and the unstructured region after the Ig fold (p.T582K). These should all yield different effects on the protein. Lacking the rod domain, the N-terminal deletion should act like a null, though it might dominant-negatively interfere with head-to-tail assembly. The rod p.E358K mutation has been tested before, yielding conflicting results in assembly studies with one reporting no disruption of filaments and the other reporting deficient assembly *in vitro*, more soluble protein in the nucleoplasm and reduced mechanical stability [39], [40]. In contrast the Ig fold mutation is on the surface but with the backbone buried so that it should still enable the beta sheet, that it is a part of, to form, but push it out relative to the adjacent beta sheet. p.R545C is in a basic patch and so might change charged interactions and p.T582K is hard to predict as it is in an unstructured region.

Other studies showing redistribution used mutant over-expression in tissue culture cells of the L530P and R377H mutations [33], [34], which may have influenced results. In these cases the cells used were mouse embryonic fibroblasts (MEFs), lymphoblastoid cell lines and standard cancer cell lines as opposed to the myoblast/fibroblast cultures, myotubes and patient muscle tissue sections used here. In the study where the emerin g.631delTCTAC mutation and nesprin 2β T89M and 1α V572L/2β T89M combined mutations were found to respectively affect the localisation of the other protein [13] patient cells and muscle sections were used; however, muscle sections were presumably only available from the patient with the combined nesprin 1α V572L/2β T89M mutations that exhibited a more striking phenotype in cultured cells than other individual mutations tested in their study. Nonetheless, in keeping with their results, we did find more intense relative nesprin 1 staining in the ER in the patient with an emerin mutation. However, for the EDMD-linked proteins we found that none exhibited a consistent redistribution phenotype throughout the wider collection of patient mutations analysed here. Moreover, by analysing a wider range of controls than most other studies, we observed considerable variation within the control population that was as strong or stronger than that observed for all NET stainings except for that of nesprin 1 and SUN2.

It is noteworthy also that many of the reports using over-expressed mutant proteins in cancer cell lines or dermal fibroblasts in culture highlighted defects in nuclear morphology and blebbing. In contrast, here using patient myoblast/fibroblast cultures and myotubes at relatively early passage number and skeletal muscle sections we observed very little nuclear morphology defects or blebbing. This argues that aspects of 2-dimensional tissue culture, rapidly dividing cancer cell lines and senescence of dermal fibroblasts probably underlie these phenotypes. Such changes, particularly senescence, could also have influenced previous reports of aberrant distribution of EDMD-linked proteins.

While we did not observe notable shared differences for any of the EDMD-linked proteins, we did observe many differences for the muscle-specific NETs in myotubes. In tissue culture these tended, like the nesprin 1 and SUN2 effects, to be observed only in distinct subsets of patient cells. The redistribution of Tmem38A to the sarcoplasmic reticulum was observed in all but one of the patient *in vitro* differentiated myotubes and was observed in all patient skeletal muscle tissue sections, though it was not sufficiently striking to be used effectively diagnostically. Differences were also observed in both *in vitro* differentiated myotubes and muscle tissue sections for Tmem214 and NET5/Samp1, though as for nesprin 1 and SUN2 these were only observed in subsets of patients.

NET5/Samp1 is particularly interesting because it also interacts with lamin B1 and SUN1 [41] and its mutation affects the distribution of SUN1, emerin and lamin A/C [42]. Samp1 also associates with TAN-lines that are important for nuclear migration [43]. This provides it with a function that could underlie the pathology of the disorder and a molecular network that parallels that of the nesprins [44], [45]. WFS1 and Tmem38A are also interesting because they are important for proper muscle gene expression and for muscle differentiation [27]. In fact, disruption of three muscle-specific NETs participating in this function together almost completely blocked myogenesis, though knockdown of each alone had little effect [27]. Thus, these NETs are prime candidates to mediate EDMD pathology because muscles appear to develop normally and then exhibit defects when they begin to be more heavily used, *i.e.* gene expression defects that prevent the muscle from fully functioning make for a reasonable explanation of pathophysiology. Tmem38A could also influence Ca^2+^ regulation [28], [29], [30], [31], especially considering its increase in the sarcoplasmic reticulum relative to that in the nuclear envelope in patients compared to controls. Though much still needs to be done to prove their participation in EDMD pathophysiology, the finding of stronger redistribution effects for these muscle-specific NETs across a panel of EDMD patient mutations than for that in the already linked proteins raises the strong possibility of their involvement as new players in EDMD.

## 5 Conclusions

Taken together this might suggest the hypothesis that the clinical variability of EDMD is also mirrored on a cellular level. Although we do not have sufficient clinical details to make a clear statement about this, it is interesting that patient P3 was reported to have a mild clinical severity score (mild weakness and rigidity only) and cells from this patient exhibited no relative increase in cytoplasmic staining compared to that in the nuclear envelope for any antibody compared to controls except for nesprin 1 (Fig. 1, Fig. 2, Fig. 4). However, patients P1, P2, P4 and P5 were all graded as moderate severity (moderate weakness and atrophy, rigidity and contractures) and variously exhibited distribution defects with between 2 and 6 different antibody stainings. Thus more work will be needed to determine if a specific mutation and distribution defect could be diagnostic of severity or clinical progression. Several different proteins at the NE can be affected to varying degrees, yet many of them exhibit interactions that suggest their co-functioning in a larger network. In addition to WFS1 and Tmem38A co-functioning in myogenic genome regulation and the NET5/Samp1 partners, redundancy of functions is observed for emerin and MAN1 and for SUN1 and SUN2 [46], [47]. That we show several different NETs can be affected to varying degrees in EDMD muscle further clarifies EDMD as a NE disorder and indicates that many different pathways to disrupt NE organisation yield a similar muscle phenotype.

## Funding

This work was supported by an MRC PhD studentship to P.L.T., Wellcome Trust Senior Research Fellowship 095209 to E.C.S. and the Wellcome Trust Centre for Cell Biology core grant 092076.

## Figures and Tables

**Table 1:** Patient and control myoblast/fibroblast cultures used in this study.

	Type	Gender	Age at biopsy (years)	Source
C1	Control	Female	36	MTCC
C2	Control	Male	35	MTCC
C3	Control	Male	5	CNDB
P1	FHL1	Male	51	MTCC
P2	LMNA p.R453W	Female	12	MTCC
P3	EMD p.Y59*	Female	17	MTCC
P4	LMNA p.R545C	Male	18	MTCC
P5	Unknown	Male	In teens	MTCC
P6	LMNA p.T582K	Male	2	CNDB
P7	LMNA p.E358K	Female	2	CNDB

**Table 2:** Tissue sections used in this study.

	Type	Gender	Age at biopsy (years)	Source
C4	Control	Female	14	CIND, Oswestry
C5	Control	Male	3	CNDB
P6	LMNA p.T582K	Male	10	CNDB
P7	LMNA p.E358K	Female	2	CNDB
P8	LMNA p.E31del	Female	2	CNDB
P9	*LMNA* de novo in exon 3	Female	5	CNDB

**Table 3:** Primary antibodies used in this study.

Antigen	Host	IF dilution	WB dilutions	Band size	Source
Skeletal myosin (FAST)	Mouse	1:50	N/A	200 kDa	Sigma (M1570) clone My-32
Lamin A/C	Rabbit	1:50	1:1000	70 kDa	Schirmer et al., 2001 (3262)
Tmem38A	Rabbit	1:50	1:200	30 kDa	Millipore (06-1005)
WFS1	Rabbit	1:50	1:200	100 kDa	Proteintech (11558-1-AP)
Tmem214	Rabbit	1:50	1:200	70 kDa	Proteintech (20125-1-AP)
NET5	Rabbit	1:20	1:100	70 kDa	Millipore (06-1013)
Dystrophin	Mouse	1:50	N/A	271 kDa	Glenn Morris (MANDYS1 (3B7))
Emerin	Mouse	1:50	N/A	29 kDa	Glenn Morris (MANEM1 (5D10))
Nesprin1	Mouse	1:50	N/A	N/A	Glenn Morris (MANNES1E (8C3))
Nesprin2	Mouse	1:50	N/A	N/A	Glenn Morris (MANNES2A (11A3))
Lamin A/C	Mouse	1:50	N/A	70 kDa	Glenn Morris (MANLAC1 (4A7))
SUN1	Rabbit	1:50	N/A	N/A	Atlas antibodies (HPA008346)
SUN2	Rabbit	1:50	N/A	N/A	Millipore (06-1038)
FHL1	Rabbit	1:50	N/A	32 kDa	Aviva Systems Biology (ARP34378_T100)
GFP	Rabbit	N/A	1:200	25 kDa	Generated in Schirmer Lab to whole protein
GFP	Mouse	N/A	1:1000	25 kDa	Clontech (632381)
H3	Mouse	N/A	1:200	17 kDa	Abcam (10799)
